# Characteristics of Stem Cells Derived from the Degenerated Human Intervertebral Disc Cartilage Endplate

**DOI:** 10.1371/journal.pone.0026285

**Published:** 2011-10-18

**Authors:** Lan-Tao Liu, Bo Huang, Chang-Qing Li, Ying Zhuang, Jian Wang, Yue Zhou

**Affiliations:** Department of Orthopedics, Xinqiao Hospital, Third Military Medical University, Chongqing, People's Republic of China; Brunel University, United Kingdom

## Abstract

Mesenchymal stem cells (MSCs) derived from adult tissues are an important candidate for cell-based therapies and regenerative medicine due to their multipotential differentiation capability. MSCs have been identified in many adult tissues but have not reported in the human intervertebral disc cartilage endplate (CEP). The initial purpose of this study was to determine whether MSCs exist in the degenerated human CEP. Next, the morphology, proliferation capacity, cell cycle, cell surface epitope profile and differentiation capacity of these CEP-derived stem cells (CESCs) were compared with bone-marrow MSCs (BM-MSCs). Lastly, whether CESCs are a suitable candidate for BM-MSCs was evaluated. Isolated cells from degenerated human CEP were seeded in an agarose suspension culture system to screen the proliferative cell clusters. Cell clusters were chosen and expanded *in vitro* and were compared with BM-MSCs derived from the same patient. The morphology, proliferation rate, cell cycle, immunophenotype and stem cell gene expression of the CESCs were similar to BM-MSCs. In addition, the CESCs could be induced into osteoblasts, adipocytes, chondrocytes, and are superior to BM-MSCs in terms of osteogenesis and chondrogenesis. This study is first to demonstrate the presence of stem cells in the human degenerated CEP. These results may improve our understanding of intervertebral disc (IVD) pathophysiology and the degeneration process, and could provide cell candidates for cell-based regenerative medicine and tissue engineering.

## Introduction

Low back pain is one of the most common reasons for seeking medical advice and is a major cause of work-related disabilities [1]. The most common cause of low back pain is degenerative disc disease (DDD) [2]. Many factors have been believed to influence the intervertebral disc (IVD) degeneration, including mechanical loading [3] and hereditary factors [4]. A possible role of the intervertebral cartilage endplate (CEP) has also been pointed out [5]. The CEP is a thin layer of hyaline cartilage that separates the vertebral bone from the disc and prevents the nucleus pulposus (NP) from bulging into the adjacent vertebrae. Changes in biochemical composition within the CEP are related closely to DDD. Proteoglycan molecules are critical for the control of solute transport through the disc, and the depletion of proteoglycans from the CEP is associated with loss of proteoglycans from the NP [6]. Thus, proteoglycan loss within the CEP will ultimately lead to DDD [7]. The disc is regarded as the largest avascular structure in the body, and mature discs are almost totally reliant on the diffusion of nutrient solutes across the CEP for metabolic exchange [8]. Calcification of the CEP or sclerosis decreases the permeability of glucose, oxygen, and other amino acids into the disc, and this finally lead to DDD [9], [10]. However, the onset of CEP calcification is not well understood [11], [12]. Apoptosis of chondrocytes within the CEP is another main cause of DDD [13]. According to some authors, CEP degeneration plays a crucial role in the initiation and development of DDD [14]. Therefore, studies of cell composition and bionomical characteristics are crucial to clarify the mechanisms of CEP degeneration and DDD.

Current treatment modalities for DDD are mainly focused on providing symptomatic relief by the removal of disc tissue without treating the underlying cause or restoring mechanical function. Therefore, therapies that both alleviate painful symptoms and restore disc structure and mechanical function by directly addressing the underlying biological causes of disc degeneration are needed [15]. Recently, cell-based therapies for regenerating or repairing the disc have become treatment options [15], [16].

Therapies based on mesenchymal stem cells (MSCs), such as tissue engineering, have gained much public interest. Human bone marrow MSCs (BM-MSCs), which were firstly isolated in 1999 by Pittinger, are easy to collect and are considered to be a promising cell source for various tissue repair and regeneration [17]. In addition, a number of adult tissues (adipose, muscle, tendon, synovial membrane, skin, IVD, and periosteum) contain populations of stem cells [18]–[20]. All of these MSCs share similar characteristics, such as propagation in culture for many generations, self-renewal ability, and capacity for multilineage differentiation. These characteristics make them suitable candidates for cell-based treatment of DDD.

Current cell-based therapies mainly target NP degeneration, but calcification of the CEP may block nutrient supply to the implanted cells and finally affect the cell activity [11], [12]. Therefore, the state of CEP plays an important role for the success of cell-based therapies. Stimulating the in situ cells within the degenerated CEP to proliferate and synthesize the matrix may be an ideal approach to prevent its degeneration and to maintain the activity of the implanted cells. Studies to clarify the cell characteristics of degenerated CEP and to determine whether stem cells exist within this tissue are meaningful.

In the present study, we isolated stem cells from degenerated human CEP harvested through posterior discectomy procedure for the lumber degenerative disease, and compared them with BM-MSCs obtained from the same patient. Both types of cells had similar morphology, proliferation rate, cell cycle, immunophenotype, and stem cell gene expression. CESCs could be induced into osteoblasts, adipocytes, and chondrocytes, and they are superior in terms of osteogenesis and chondrogenesis. These data indicate that CESCs may be a potential candidate for cell-based regenerative medicine and tissue engineering.

## Materials and Methods

### Ethics Statement

Institutional Review Board approval and informed consent for sample collection were obtained before collection of all samples. All the procedures specified below were approved by the Ethical Committee of Xinqiao Hospital and were in accordance with the Helsinki Declaration.

### Case Selection

The CEP used in this study was obtained from 7 patients who underwent the posterior discectomy procedure for the lumbar degenerative disease. During the surgical procedure, 5–10 ml bone marrow (BM) was obtained by iliac crest aspiration from the same patient. The average age was 42.1 years (range: 36–50 years), and the male/female ratio was 3/4. Details of all samples were shown in Table 1.

**Table 1:** Details of the patients enrolled in this study.

Case No.	Diagnosis	Disc Level	Modic Type	BM(ml)	Gender	Age(year)
1	**Spondylolisthesis**	**L4-L5**	**II**	7.0	M	40
2	**Spondylolisthesis**	**L4-L5**	**I**	6.5	F	39
3	**Spondylolisthesis**	**L5-S1**	**II**	10.0	M	36
4	**Spinal stenosis**	**L5-S1**	**II**	5.0	F	50
5	**Vertebral instability**	**L4-L5**	**II**	7.5	F	43
6	**Discogenic low back pain**	**L5-S1**	**II**	6.5	M	46
7	**Discogenic low back pain**	**L4-L5**	**I**	8.5	F	41

### Isolation and Culture of Human BM-MSCs

BM-MSCs were isolated as previously described [21]. Briefly, 5–10 ml of BM aspirate was collected in a syringe containing 10,000 IU of heparin to prevent coagulation. Low-density mononuclear cells were isolated by centrifugation through 1.073 g/ml of Percoll solution (Sigma, USA) at 1000×g for 30 minutes. The cells were rinsed twice with phosphate buffered saline (PBS) and resuspended in Dulbecco's modified Eagle's medium/F12 (DMEM/F12, HyClone, USA) supplemented with 10% fetal calf serum (FCS, HyClone, USA) and 100 U/ml penicillin-streptomycin (HyClone, USA). All of the nucleated cells were cultured in a 25-cm^2^ cell culture flask (Costar Corning, USA) in a humidified atmosphere containing 5% CO_2_ at 37°C. After 24 hours, the nonadherent cells were removed by replacing the medium, and the adherent cells were washed twice with PBS and cultured with above culture medium. After reaching 90% confluence, the cells were harvested with 0.25% trypsin and 1 mM EDTA (trypsin/EDTA, HyClone, USA) for 3 minutes at 37°C, and subcultured at a dilution of 1∶3. Passage 3 cells were used in the study.

### Isolation and Culture of Degenerated Human CEP-derived Cells

Surgically explanted CEP was cleaned of any adherent extraneous invasive tissue under the sterilized dissecting microscope (Leica, Germany).Briefly, remove the NP, annulus fibrosus and subchondral bone tissues around blocks of CEP using ophthalmic operating set under dissecting microscope (4× magnification). The average thickness of CEP blocks was 0.8 mm. After washing with PBS, a small tissue (from each donator) was used for hematoxylin and eosin staining (HE) to rule out whether any other tissues were also present in the CEP. The remaining sample was minced into pieces no larger than 1 mm^3^ and digested with 0.15% collagenase II [Sigma, USA] in DMEM/F12 containing 3% FCS for 12 hours at 37°C. At the end of the digestion, the suspended cells were filtered through a 70-µm cell filter to minimize cell aggregates. The cell suspension was transferred to a sterile conical tube and centrifuged for 10 minutes at 200×g. After aspirating the supernatant, the pellet was resuspended in DMEM/F12 supplemented with 10% FCS and 5 units/ml penicillin and streptomycin. Total cell number per tissue sample was 2.5×10^5^ to 7.5×10^5^. Then the cells were cultured in 25-cm^2^ cell culture flask in a humidified atmosphere containing 5% CO_2_ at 37°C. After the first passage of expansion, cells were sub-cultured in agarose suspensions.

### Agarose Cultures to Select CESCs

The agarose culture was established using the protocol published by Thornemo in 2005 [22]. Briefly, 2% low-melting point agarose (Invitrogen, USA) was sterilized by autoclaving andthen equilibrated to 37°C before the next procedure. Culture dishes (60 mm in diameter, Costar Corning, USA) were coated with 1% low-melting point agarose which was mixed with an equal volume of 37°C 2× DMEM/F12 and 2% low-melting point agarose and the excess agarose was removed by aspiration.Subsequently, a mixture of 0.75 ml of DMEM/F12, 0.75 ml of 2% low-melting point agarose and 1.5 ml of 20% FCS DMEM/F12 containing 5×10^4^ P1 CEP-derived cells was added to the culture dishes. The final concentration of FCS was 10%. Culture dishes were held at 4°C for 15 minutes until the gels solidified. The culture dishes were then incubated in a humidified atmosphere containing 5% CO_2_ at 37°C. Change culture medium with DMEM/F12 supplemented with 10% FCS and 5 units/ml penicillin and streptomycin twice a week. After 6 weeks, cell clusters (diameter greater than 50 µm) were isolated using a sterile Pasteur pipette and subcultured in multi-well plates (Costar Corning, USA). Passage 3 CESCs were used in the study.

### Measurement of Cell Proliferation Capacity

To measure the proliferation capacity of the CESCs and BM-MSCs, Cell Counting Kit-8 (CCK-8, Dojindo Laboratories, Japan) was used as previously described [23]. Briefly, 10 µL of CCK-8 solution was added to each well which contained 3000 cells. After incubating the 96-well plate at 37°C for 4 hours, the absorbance at 450 nm was measured using a microplate absorbance reader (BioRad, USA). Cell proliferation was tested on days 1, 3, 5, 7 and 9. The blank 96-well plate was used for the zero setting. All experiments were performed in triplicate.

### Cell Cycle Assay

For the cell cycle analysis, both CESCs and BM-MSCs were fixed with 75% cold ethanol for 72 hours at 4°C. After washed with PBS two times, 1 ml propidium iodide (PI, Invitrogen, USA) staining solution and 50 µL RNase A stock solution (Invitrogen, USA) were added and incubated at 4°C for 3 hours. Measurements were performed on a FACSCalibur flow cytometer (BD, USA) by using CellQuest software (BD, USA) and acquiring ≥20,000 cells per sample.

### Determination of the Cell-surface Antigen Profile

For the immunophenotypic characterization of BM-MSCs and CESCs, cells were washed with PBS and incubated with the following fluorescein isothiocyanate (FITC)-, phycoerythrin (PE)-, peridinin chlorophyll protein (PerCP)-, allophycocyanin (APC)- coupled monoclonal antibodies: CD14-FITC, CD19-APC, CD34-FITC, CD45-FITC, CD44-PE, CD73-FITC, CD90-FITC, CD105-PE, CD133-FITC, CD166-FITC, HLA-DR-PerCP and STRO1-FITC. All antibodies were purchased from eBioscience (USA), except for STRO1-FITC (Santa Cruz Biotechnology, USA). Isotype control antibodies were used in each case. After incubating for 30 minutes at 37°C, cells were washed 3 times with PBS. Finally, labelled cells were re-suspended in 500 µl PBS and subjected to single channel flow cytometry analysis (BD, USA). At least 10^4^ events were collected per sample. The extent of positive staining was calculated as a percentage in comparison with the isotype staining.

### Stem Cell Gene Expression Assay

To evaluate the expression of the genes responsible for stem cells, three stem cell genes (*NANOG*, *SOX-*2 and *OCT-4*) were analyzed using the reverse transcriptase polymerase chain reaction (RT-PCR). Details of the related genes were listed in Table 2, and the procedure was depicted below.

**Table 2:** Primers used for RT-PCR and qPCR analysis.

Target Gene	Primers Sequence	T_A_(°C)	Cycles
RUNX-2	sense 5′-ACGACAACCGCACCATGGT-3′antisense 5′-CTGTAATCTGACTCTGTCCT-3′	59	30
alkaline phosphatase (ALP)	sense 5′-TGGAGCTTCAGA AGCTCAACACCA-3′antisense 5′-ATCTCGTTGTCTGAGTACCAGTCC-3′	59	28
osteocalcin (OC)	sense 5′-ATGAGAGCCCTCACACTCCTC-3′antisense 5′-GCCGTAGAAGCGCCGATAGGC-3′	59	30
peroxisome proliferators-activated receptor 2 (PPAR-2)	sense 5′-CGAGGGCGATCTTGACAGGAA -3′antisense 5′-CAGGGGGGTGATGTGTTTGAAC- 3′	55	30
adipogenic protein (APP)	sense 5′-CTGTCCAAGTCCAACAGCAA-3′antisense 5′-ACGTTGGCAGCTTTACGTCT-3′	55	30
lipoprotein lipase (LPL)	sense 5′-TCCGCGTGATTGCAGAGAGAG-3′antisense 5′-TGCTGCTTCTTTTGGCTCTGACT-3′	55	30
aggrecan (Agg)	sense 5′-TGAGGAGGGCTGGAACAAGTACC-3′antisense 5′-GGAGGTGGTAATTGCAGGGAACA-3′	57	28
collagen II (Col II)	sense 5′-TTTCCCAGGTCAAGATGGTC-3′antisense 5′-TCACCTGGTTTTCCACCTTC-3′	57	30
SOX-9	sense 5′-TGGCCGAGATGATCCTAAAAATAA -3′antisense 5′-GCGCTTGGATAGGTCATGTTTGT-3′	57	30
SOX-2	sense 5′-CCCCTGTGGTTACCTCTTCCTC-3′antisense 5′-GGCCGCTCTGGTAGTGCTG-3′	61	32
NANOG	sense 5′-ACCCCGTTTCACTGTGTTAGC-3′antisense 5′-GACGGCAGCCAAGGTTATTAAA-3′	63	32
OCT4	sense 5′-GGCAAGCGATCAAGCAGCGAC-3′antisense 5′-GGGAAAGGGACCGAGGAGTAC-3′	61	32
β-actin	sense 5′-GTGGGGCGCCCCAGGCACCA-3′antisense 5′-CTTCCTTAATGTCACGCACGATTTC-3′	55	45

### Differentiation Procedures and Related Quantitative Assays

For the *in vitro* differentiation assays, three procedures were used.

#### (1). Osteogenic Induction

The cells were incubated in an osteogenic medium containing 10% FCS, 10 nM dexamethasone, 10 mM β-glycerophosphate, and 0.1 mM L-ascorbic acid-2-phosphate for 3 weeks. The medium was changed twice a week. Negative control wells were maintained in DMEM/F12 supplemented with 10% FCS.

### Quantitative Assay of Mineral Deposits

1.5×10^4^ cells were seeded in each well of a 24-well plate. The cells were incubated in the osteogenic medium for 1, 2, 3 or 4 weeks. After each induction time, the cells were washed with PBS and fixed in 70% ethanol for 1 hour at room temperature. Then the cells were stained with 40 mM/L alizarin red (ph 4.2) for 10 minutes at room temperature. After washing with distilled water 5 times, the cells were incubated with PBS at 37°C for 15 minutes. Finally, the cells were incubated with 300 µl 10% CPC (cetylyridinium chloride) at 37°C for an additional 15 minutes and the optical density of the extracted dye was evaluated at 562 nm in a spectrophotometer.

### Quantitative Assay of Alkaline Phosphatase (ALP) Activity

To quantify ALP activity, a modified procedure was used as described previously [24]. Briefly, 1.5×10^4^ cells of the BM-MSCs and CESCs were seeded in each well of a 24-well plate, and incubated in the osteogenic medium for 1, 2, 3 or 4 weeks. After each induction time, the cells were washed with PBS and incubated in 400 µl lysed solution with 10 mM Tris-HCl containing 1 mM MgCl_2_ and 1% Triton X-100 at 4°C. Subsequently, 50 µl (6.7 mM/L) fresh freezing p-nitrophenyl phosphate (pNPP) was used as the substrate to measure ALP activity based on p-nitrophenol (pNP) release. The substrate and the lysed solution were incubated at 37°C for 2 hours. Finally, the reaction was stopped by the addition of 100 µl 0.1 M NaOH and was monitored at 405 nm. The optical density of the extracted dye was evaluated at 562 nm in a spectrophotometer.

#### (2). Adipogenic Induction

Adipogenic differentiation was performed in cultures with induction and maintenance medium. Briefly, these cells were exposed to an induction medium containing 10 µg/ml insulin, 1 µM dexamethasone, 500 µM 3-isobutyl-1-methyl xanthine, and 100 µM indomethacin for 72 hours. Then the medium was replaced with maintenance medium containing 10 µg/ml insulin in DMEM and 10% FCS and cultured for an additional 24 hours. This 96-hour treatment cycle was repeated three times, followed by culture for several additional days in adipogenic maintenance medium until 21 days. Negative control wells were maintained in DMEM/F12 supplemented with 10% FCS for the duration of the assay. To visualize the lipid-rich vacuoles in the cells, cells were stained with oil red o. Briefly, cell layers were fixed with 4% formaldehyde and stained with oil red o for 15 minutes. Hematoxylin was used for nuclear staining. Finally, the cultures were extensively washed with water to remove excess stain, and were observed by microscopic inspection (Leica, Germany). To quantify adipogenic differentiation by morphology, the differentiation value (DV) method was adopted as previously described [25]. Briefly, 105 high power fields (15 random fields from one source, 200×magnification ) from 7 biological sources of BM-MSCs and CESCs were counted using Image-Pro Plus version 6.0 (IPP 6.0). The DV was determined by dividing the lipid droplet area by the number of nuclei.

### Quantitative Assay of Adipogenic Capacity

To quantify the adipogenic capacity of BM-MSCs and CESCs, quantitative adipogenesis kit (Genmed, USA) was used. This kit used a colorimetric assay kit that was based on oil red o staining the lipid-rich vacuoles red. The optical density (OD) increased with the appearance of more red lipid-rich vacuoles in the cells. The OD values indicated the adipogenic capacity. The procedure was performed strictly according to the manufacturer's instruction. Briefly, 1.5×10^4^ BM-MSCs and CESCs were seeded in each well of a 96-well plate. After incubated at 37°C for 90 minutes, adipogenic induction medium was added into each well and cultured for 1, 2 or 3 weeks. At each induction time point, the wells were cleaned, fixed, dyed with oil red o, lysed on a shaking table at 50 reverse per minute at 37°C for 30 minutes according to the instruction. The density of the extracted dye was evaluated at 490 nm in a BioRad reader. The blank 96-well plate was set as the zero level.

#### (3). Chondrogenic Induction

Cells were incubated using the micromass culture method. Micromass cultures were generated by adding 20 µl droplets of cell solution (1.0×10^7^cells/ml) in the center of multi-well plate wells. After cultivating the micromass cultures for 2 hours under high humidity conditions, the chondrogenic medium, which contained low-glucose DMEM, supplemented with 10 ng/ml transforming growth factor (TGF)-β_3_, 10^−7^ M dexamethasone, 50 µg/ml ascorbate-2-phosphate, 40 µg/ml proline, 100 µg/ml pyruvate, and 1∶100 diluted ITS+Premix, was added to the culture and incubated up to 3 weeks. The medium was changed twice per week.

### Quantitative Assay of Alcian Blue Intensity

The alcian blue intensity assay was performed as described previously [26]. Briefly, both cells were cultured in the micromass culture for 1, 2 or 3 weeks. After each time point, the cells were rinsed twice with PBS, fixed with 10% neutral buffered formalin (pH 7.4) including 1% CPC for 15 minutes at room temperature, then washed with PBS, and covered with 1% alcian blue 8 GS (pH 1.0 ) in 0.1 M HCl for 1 hour. The cultures were rinsed with 50 mM Tris-HCl (pH 7.4) and incubated with 300 µL solution that containing 4 M guanidine–HCl, 50 mM Tris–HCl, 0.1% CHAPS( pH 7.4) for 3 hours at room temperature. The optical density of the extracted dye was evaluated at 595 nm in a spectrophotometer.

### Reverse Transcriptase Polymerase Chain Reaction (RT-PCR)

To evaluate the expression of the genes responsible for stem cells (*NANOG*, *OCT4 and SOX2*), RT-PCR was used. Total RNA was extracted from BM-MSCs and CESCs using the RNeasy kit (Qiagen GmbH, Germany) according to the manufacturer's instructions. Isolated RNA was treated with RNase-free DNase (QIAGEN GmbH) before being reverse transcribed into cDNA. Total RNA was measured by a spectrophotometer (Beckman, Fullerton, CA) at 260 nm and 280 nm. The RNA (1 µg) was reverse transcribed with an oligo (dT) primer using the ThermoScriptTM RT-PCR system (Invitrogen, USA) for cDNA synthesis. For RT-PCR, 1 µL of cDNA template was used for each reaction and sequences were amplified using Taq DNA polymerase. Stem cell gene primers were designed and synthesized as shown in Table 2. β-actin was used to normalize the reactions.

### Real-time Quantitative Reverse Transcriptase Polymerase Chain Reaction (qPCR) Assay

To compare the mRNA levels of osteogenic, adipogenic-, and chondrogenic- specific genes of BM-MSCs and CESCs after a 3-week induction, qPCR was used. Total RNA extraction was performed as described above. The RT reaction (2.0 µL) was amplified in quadruplex by real-time PCR (ABI PRISM 7000) in a final volume of 25 µL, using SYBR Green Master Mix reagent at a final concentration of 1× (Applied Biosystems, Foster City, CA). Osteogenic-, adipogenic- and chondrogenic- specific genes for qPCR were shown in Table 2. β-actin was used to normalize the reactions. For each cDNA sample, the threshold cycle (Ct) value of each target sequence was subtracted from the Ct value of the reference gene. The expression level of each target gene was calculated as 2^ΔCt^. Each sample was assessed three times for each gene. Three samples were randomly selected.

### Immunohistochemical Staining

The appropriate micromass pellet slides were deparaffinized and pretreated by heating in citrate buffer at 95–100°C for 10 minutes. The pretreated samples (5 µm thick) were then fixed in 4% paraformaldehyde, quenched with 10% H_2_O_2_ in methanol for 20 minutes, blocked with 5% normal goat serum and incubated with the following primary monoclonal antibodies in a humidified chamber at room temperature for 1 hour: type II collagen (mouse, 1∶100, Sigma, USA) and aggrecan (mouse, 1∶100, Sigma, USA). After washing with PBS, the cells were incubated with biotinylated anti-mouse IgG antibody (1∶100, Sigma, USA) in a humidified chamber at room temperature for 30 minutes. Finally, a 3, 3′-diaminobenzidine tetrahydrochloride (DAB) substrate solution was used to reveal the color of antibody staining in the presence of H_2_O_2_. The slides were rinsed in distilled water, dehydrated through graded alcohol, and mounted with p-xylene-bis-pyridinium bromide (DPX). The slides were examined using a light microscopy (Leica, Germany).

### Western Blot Assay of Type II Collagen and Aggrecan

Micromass pellets from the BM-MSCs and CESCs after 3 weeks in the chondrogenic induction medium were used for western blot analysis. The samples were incubated with an extraction solution containing 1 ml 4 M guanidine chloride (GuCl), 0.1 M -6-aminohexanoic acid, 20 mM -benzamidine hydrochloride, 10 mM -EDTA, 5 mM -N-ethylmaleimide and 0.5 mM phenylmethanesulfonyl fluoride, pH 5.0 at 4°C for 3 hours. GuCl extracted dialysate was resuspended in 50 ml of sample buffer and analyzed by electrophoresis on a 6% (w/v) SDS-PAGE gels. Equal amounts of protein per lane (50 µg) were loaded and separated by electrophoresis. Protein was transferred to a polyvinylidene fluoride (PVDF) membrane by electroblotting. Rinse the membrane in water and block the blotted membrane in freshly prepared PBS containing nonfat dry milk (5%) for 60 minutes at room temperature with constant agitation. The membrane was incubated with the human type II collagen (mouse, 130 kD, 1∶1000, Sigma, USA) and aggrecan (mouse, 250 kD, 1∶1000, Sigma, USA) antibodies overnight at 4°C with agitation. After washed the membrane three times with PBS, the secondary goat anti-mouse horseradish peroxidase (HRP)-conjugated antibody was added and incubated overnight at 4°C with agitation. Subsequently, the blots were washed 5 times with PBS containing 0.05% Tween 20 and the signal of bound antibodies was developed by enhanced chemiluminescence.

### Histochemical Staining

#### Alizarin red staining

To identify the mineral deposits by alizarin red staining, the cells in culture medium, were fixed in 70% ethanol for 10 minutes and stained with 0.5% alizarin red (pH 4.1) for 10 minutes.

#### Oil red o staining

To localize lipid droplets, cell layers were fixed in 4% paraformaldehyde for 30 minutes and incubated in oil red o solution for 15 minutes. Finally, the cultures were extensively washed with water to remove excess stain, and microscopically observed.

#### Alcian blue staining

To visualize the deposition of sulfated glycosaminoglycans, the slides were incubated with 1% alcian blue 8GX in 0.1 M HCl overnight. Finally, cultures were washed extensively with distilled water and photographed.

### Statistical Analysis

The absorbance, percentage of cell cycle phase, immunophenotype and gene expression level was reported as mean ± SD (standard deviation). The histological and immunohistochemical data were qualitatively described. Representative images were shown. Quantifiable data comparing the BM-MSCs and CESCs were analyzed using Student's *t*-tests. All of the data analyses were performed using SPSS version 10.0. *p*<0.05 was regarded as statistically significant.

## Results

### Histomorphology of Degenerated Human CEP Tissues

**Figure 1:**
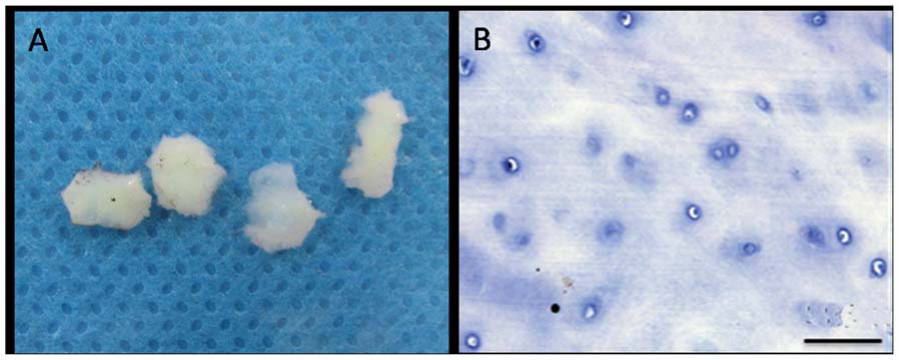
Histomorphology of degenerated human CEP tissues. A. gross morphology. B. histologic morphology with HE staining. Bar = 50 µm.

The CEP was white and translucent after being cleaned under the dissecting microscope (Figure 1A). The extracellular matrix (ECM) of CEP tissues was homogeneous and contained round-shaped chondrocytes distributed into different layers (Figure 1B). No vascular or fat tissues were observed in any of the CEP tissues.

### Morphology and Cell Proliferation Capacity of BM-MSCs and CESCs

**Figure 2:**
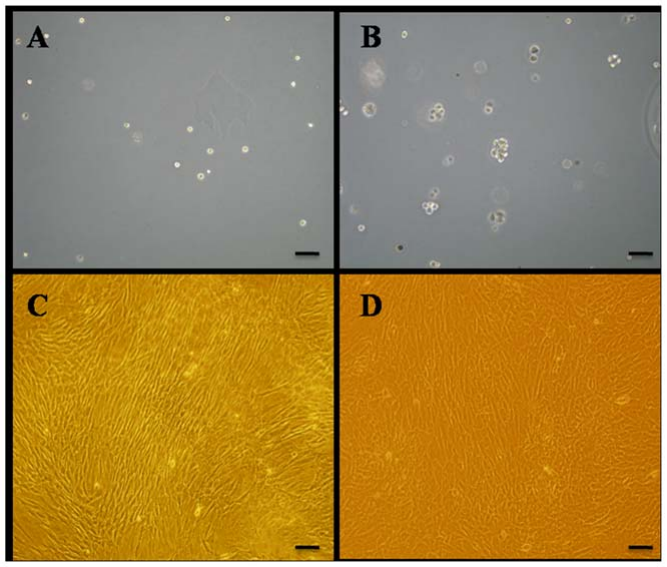
Morphology of cells derived from CEP and BM. Morphology of CEP-derived cells in agarose after immediately seeded (A) and six weeks later (B). Morphology of BM-MSCs (C) and CESCs (D). Bar = 100 µm.

**Figure 3:**
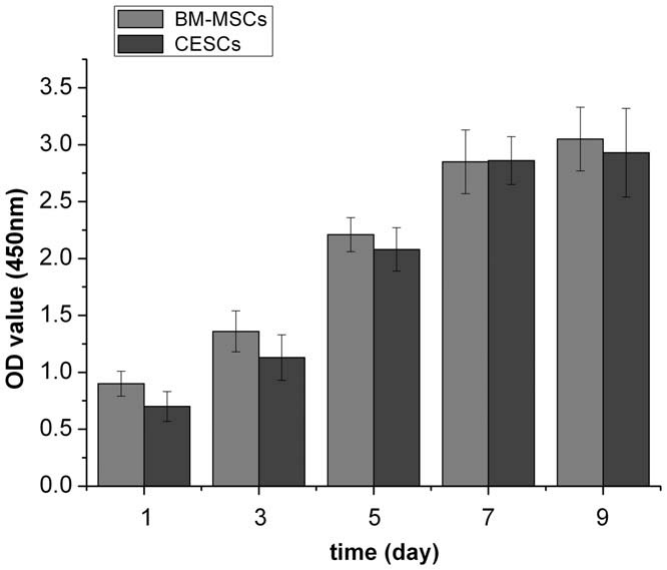
The proliferation capacity of BM-MSCs and CESCs measured using the CCK-8 assay. The OD values were not significantly different between the BM-MSCs and CESCs on day 1, 3, 5, 7 or 9, suggesting that the proliferation capacity of the two cell types were the same. Data from cells derived from 7 different patients (mean ± SD).

To screen the proliferative cells, agarose culture was used. P1 CEP-derived cells were loaded into the low melting agarose culture, and all of the cells were dispersed in the agarose and kept in monoplast suspension (Figure 2A). After being cultured in agarose for six weeks, some of the seeded cells formed cell clusters with diameters greater than 50 µm, and some cells had not divided and were still in the monoplast state (Figure 2B). We chose the cell clones with a diameter greater than 50 µm and subcultured them in multi-well plates for expansion. Mononuclear cells (5×10^5^ to 2.5×10^6^) from a 5-ml to 10-ml iliac crest aspiration were acquired from the subjects and were cultured in flasks. When P3 cells reached 90% confluence, the BM-MSCs and CESCs exhibited a homogeneous fibroblast-like morphology with a spindle shaped appearance while in culture as shown in Figure 2. Regarding the proliferation capacity, both stem cell types exhibited similar growth tendencies. The OD value increased from day 1 to day 7 and reached a plateau from day 7 to day 9. Although the OD value of BM-MSCs was slightly higher than that of the CESCs at each time point, the differences were not significant (p>0.05, Figure 3).

### Cell Cycle of BM-MSCs and CESCs

**Figure 4:**
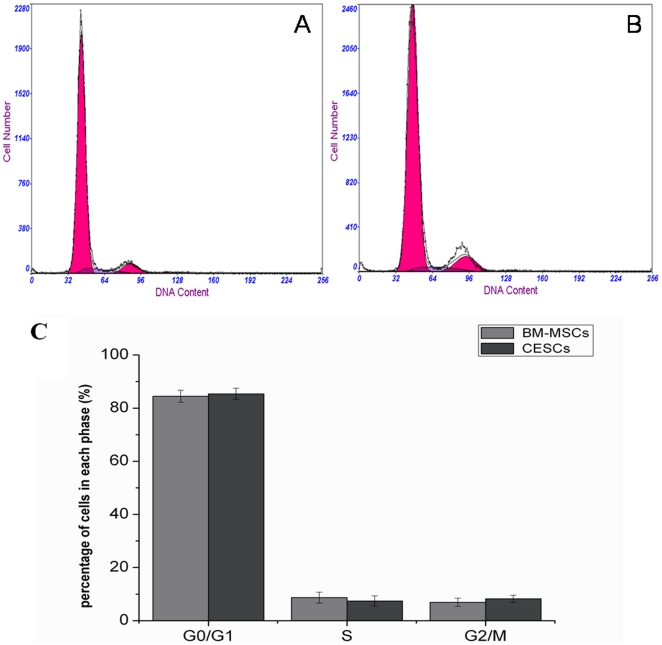
Cell cycle analysis of BM-MSCs and CESCs. Representative cell cycle graphs of BM-MSCs (A) and CESCs (B) were showed. The percentage of cells in each phase of the cell cycle was analyzed by flow cytometry (C). There were no significant differences in all cell cycle phases (G0/G1, S and G2/M) between BM-MSCs and CESCs. Data from cells derived from 7 different patients (mean ± SD).

Representative graphs of cell cycle for both stem cells were shown in Figure 4. A cell cycle analysis by measuring the DNA content revealed that a small population of the cells were engaged in proliferation (S1+G2+M<20%). More than 80% of the BM-MSCs and CESCs were in the G0/G1 phase (84.46% vs. 85.35%), and there were no significant differences in the percentage of cells in the G0/G1 phase between the BM-MSCs and CESCs. No significant differences were observed in percentage of cells in the G2/M phase (8.66% vs. 7.39%) or S phase (6.91% vs. 8.25%) between the BM-MSCs and CESCs (Figure 4C).

### Flow Cytometric Immunophenotype

**Figure 5:**
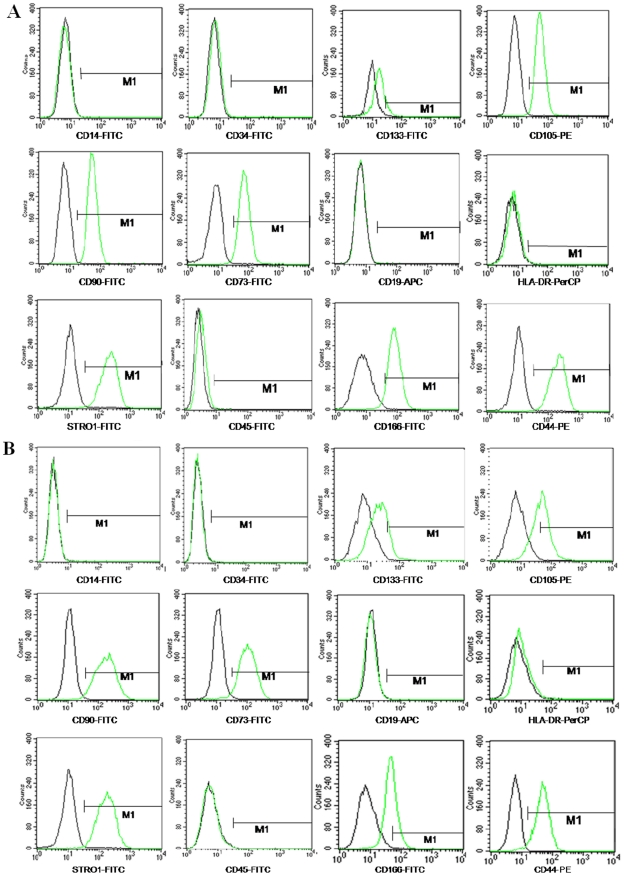
Immunophenotypic profile of BM-MSCs (A) and CESCs (B) analyzed by FACS. The green lines represent the fluorescence intensity of cells stained with the indicated antibodies and the black lines represent the negative control cells, which were stained with a non-immunoreactive isotype control antibody.

The antigenic phenotype of BM-MSCs and CESCs was checked by flow cytometric analysis (Figure 5). CESCs were positive for many stem cell markers common to BM-MSCs, including CD105, CD73, CD90, CD44, CD166 and Stro-1, and they were slightly positive for CD133. However, the percentages of CD105- and CD166- positive cells were significantly lower in the CESCs than in the BM-MSCs. The BM-MSCs and CESCs were negative for the pan-monocytic antigen CD14, the hematopoietic stem cell marker CD34, the pan-B-cell marker CD19, the pan-hematopoietic marker CD45, and the class 2 HLA antigen HLA-DR (Figure 5). The mean surface immunophenotype expression values and SD from seven donors were summarized in Table 3.

**Table 3:** The average values of surface immunophenotypes from 7 donors.

Markers	BM-MSCs mean (SD)	CESCs mean (SD)
CD73	97.34 (2.12)	96.10 (2.07)
CD90	98.45 (1.42)	98.01 (1.13)
CD44	98.87 (2.04)	96.63 (2.43)
Stro-1	98.37 (1.18)	98.04 (1.61)
CD105	97.78(2.36)	82.02(9.41)*
CD166	95.86(2.52)	45.63(12.04)*
CD133	4.63(2.73)	6.02(3.74)
CD14	0.46(0.23)	0.33(0.19)
CD34	0.65(0.18)	0.56(0.11)
CD19	0.72(0.23)	0.81(0.20)
CD45	1.12(0.67)	0.67(0.21)
HLA-DR	0.89(0.48)	0.95(0.64)

*indicated p<0.05.

### Stem Cell Gene Analysis

**Figure 6:**
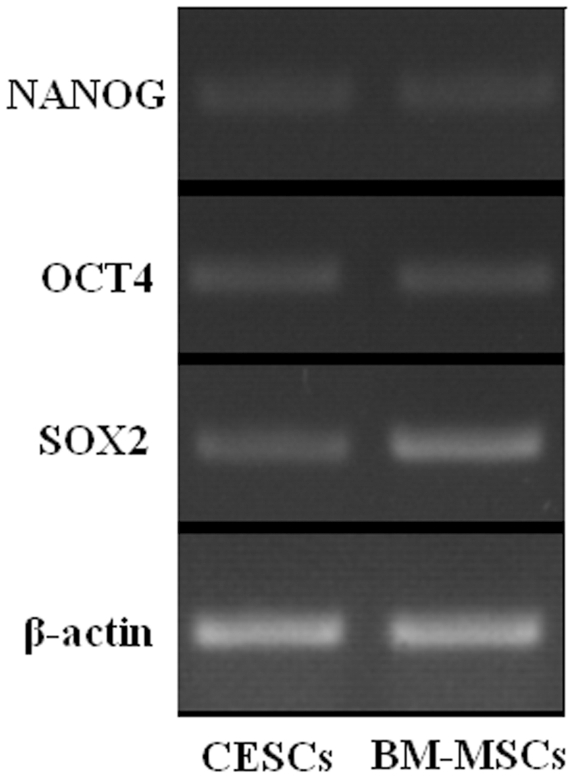
Stem cell gene expression. Stem cell genes (*OCT-4*, *NANOG*, and *SOX-2*) were expressed both in BM-MSCs and CESCs.

*OCT-4*, *NANOG*, and *SOX-2* were genes that were commonly expressed in stem cells. Passage 3 cells were chosen to identify the expression of these genes. Compared to BM-MSCs, CESCs showed comparable gene expression (Figure 6).

### Osteogenic Differentiation

**Figure 7:**
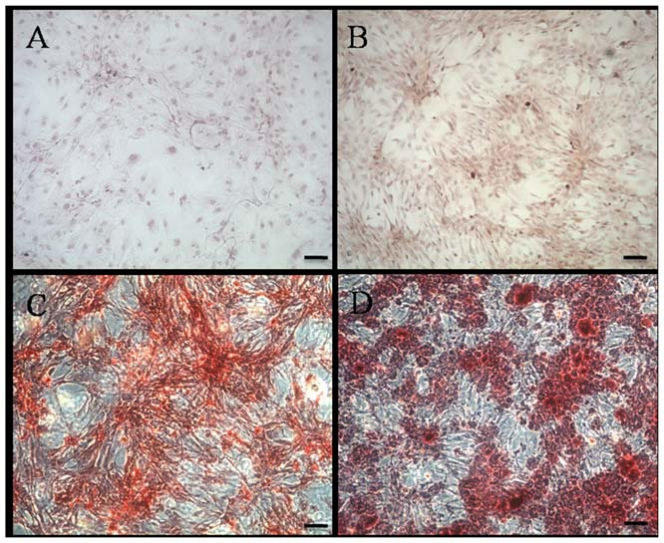
Osteogenic differentiation of BM-MSCs and CESCs stained with alizarin red after 3 weeks. BM-MSCs and CESCs treated with growth culture medium alone (A: BM-MSCs, B: CESCs) showed minimally alizarin red positive staining, and cells treated with osteogenic medium (C: BM-MSCs, D: CESCs) showed mineral deposition around the cells to form nodular aggregates after 3 weeks. Bar = 100 µm.

**Figure 8:**
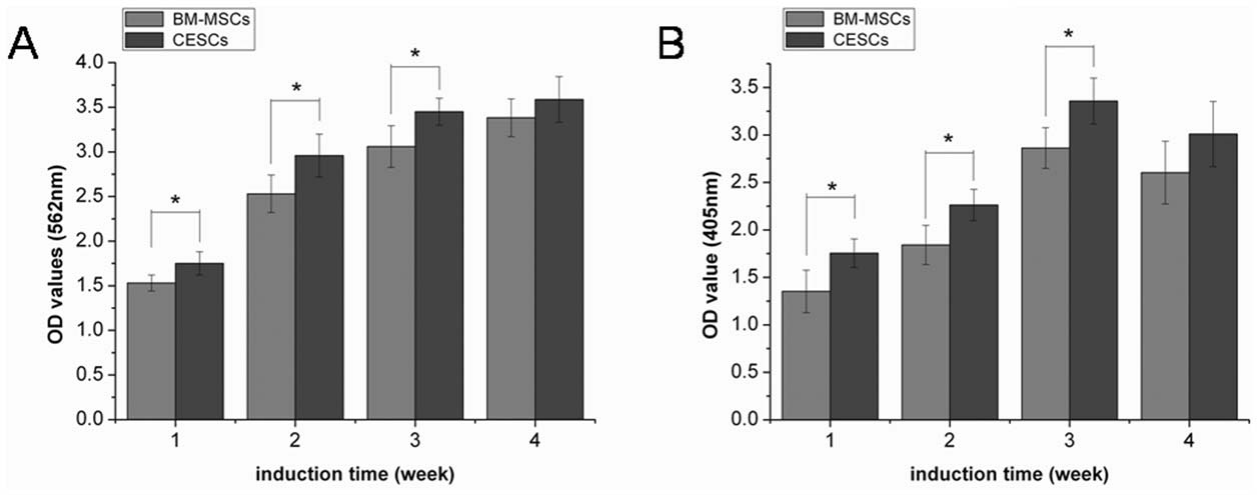
Quantitative analysis of mineral deposition and ALP activity of both cell types in the osteogenic medium. A. results of mineral deposition B. results of ALP activity. Data from cells derived from 7 different patients (mean ± SD). * indicated p<0.05.

**Figure 9:**
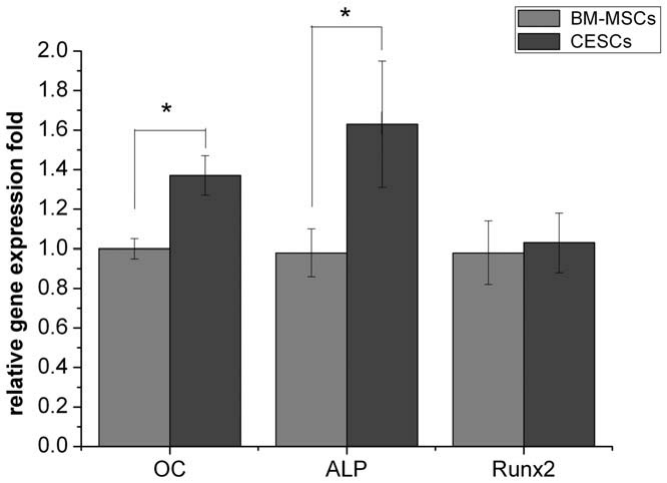
The mRNA levels of osteogenic genes. There were higher expression levels of OC and ALP after 3-week induction of CESCs compared with that of BM-MSCs. There was no significant difference of RUNX2 expression between BM-MSCs and CESCs. * indicated p<0.05.

The BM-MSCs and CESCs cultured in the growth medium for 3 weeks were negative for alizarin red (Figures 7A, B). Osteogenic differentiation was confirmed by the deposition of an alizarin red positive mineralized matrix (Figures 7C, D). From the morphology, the mineralized nodules from CESCs were larger and stained more intensely, indicating more extensive calcium deposition. Absorption of the dye at 562 nm revealed more alizarin red positive mineralized matrices from CESCs than BM-MSCs at week 1, 2, and 3, but no significant difference at week 4 (Figure 8A). The ALP activity results showed that the OD values of CESCs were greater than the OD values of BM-MSCs during the first three weeks (p<0.05), but the OD values for both decreased in the fourth week (Figure 8B). According to qPCR, no significant difference was observed in the expression of *RUNX-2* between the two groups. However, the expressions of *ALP* and *OC* were higher in CESCs (p<0.05, Figure 9). These results indicated that CESCs were superior in osteogenesis than BM-MSCs.

### Adipogenic Differentiation

**Figure 10:**
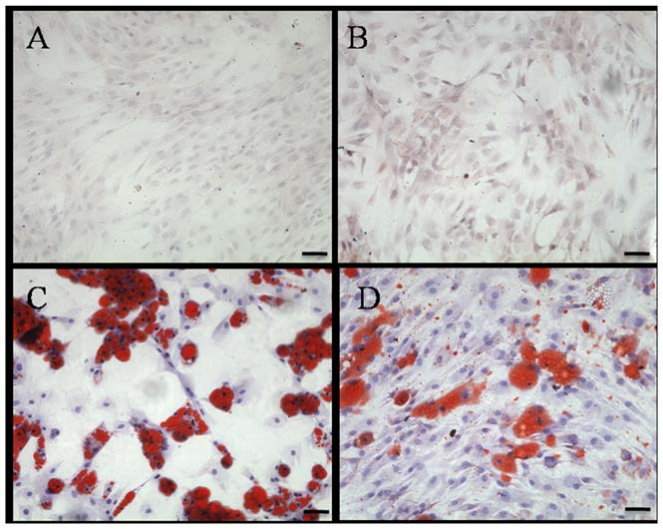
BM-MSCs and CESCs stained with oil red o after 3 week induction. BM-MSCs and CESCs were treated with growth culture medium alone (A, B) or adipogenic induction medium (C, D) and stained with oil red. Bar = 50 µm.

**Figure 11:**
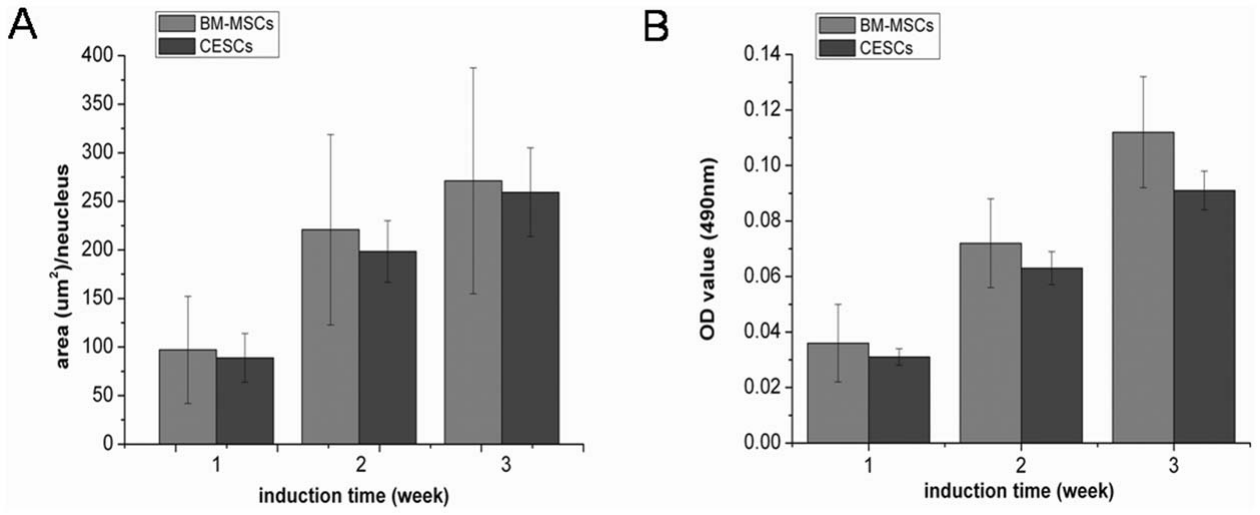
Adipogenesis of BM-MSCs and CESCs. A. Differentiation value (DV), analyzed using IPP 6.0, showed that there was no significance in DV at corresponding time point. B. Adipogenic capacity, measured using adipogenic quantitative kit at 490 nm, showed that there was no significance in adipogenic capacity at each time point. Data from cells derived from 7 different patients (mean ± SD).

**Figure 12:**
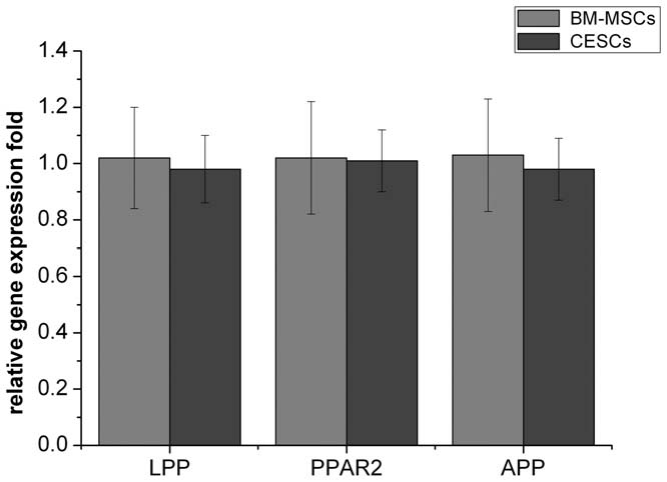
The mRNA levels of adipogenic genes after 3-week induction. There was no significant difference for all the three adipogenic genes (PPAR-2, LPP and APP) between BM-MSCs and CESCs.

The presence of lipid-rich vacuoles stained with oil red O was used to evaluate adipogenic induction. Both cell types exhibited intracellular lipid vacuoles after 3 weeks of adipogenic induction, but they were not detected in the growth medium (Figure 10). We analyzed the DV results using IPP 6.0 at each time point and found that lipid droplets gradually accumulated within both cell types, while no significant difference was found between them at any time point (p>0.05, Figure 11A). For quantitative adipogenic capacity analysis, the OD values at 490 nm showed that no significant difference existed for the adipogenic capacity between the two groups at any induction time point (Figure 11B). Regarding the expression of adipogenic genes (*PPAR-2*, *LPP* and *APP*), no significant differences were observed after 3 weeks of induction (Figure 12).

### Chondrogenic Differentiation

**Figure 13:**
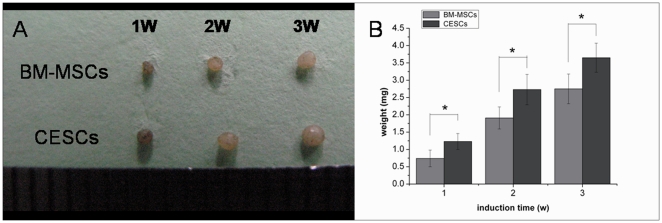
Time course for the synthesis of cartilage by BM-MSCs and CESCs cultured in micromass culture system. A. Macro pictures of differentiating aggregates from BM-MSCs and CESCs at week 1, 2 and 3. B. Wet weight of cell aggregates at week 1, 2 and 3. Data from cells derived from 7 different patients (mean ± SD, * p<0.05).

**Figure 14:**
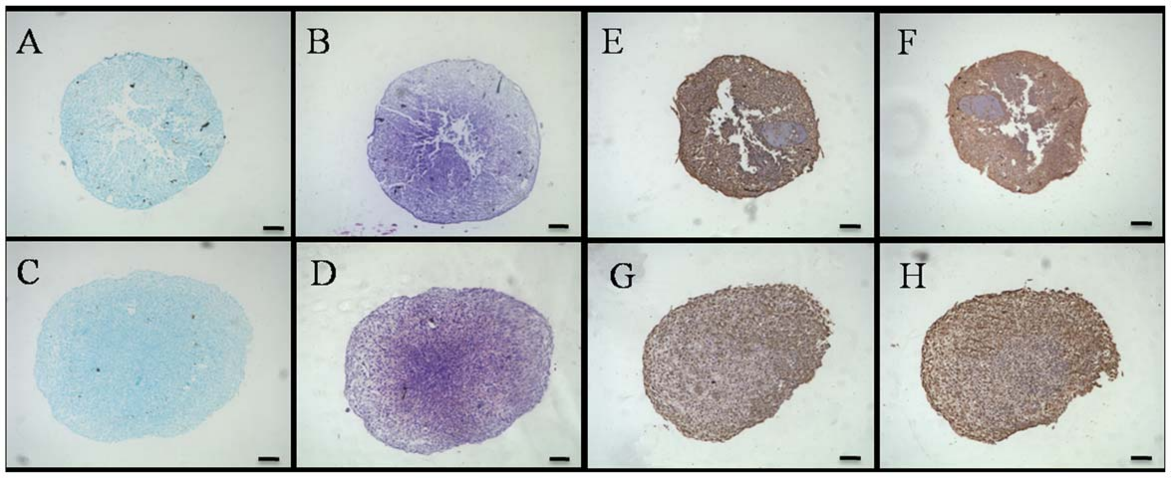
Histology of mcromass pellets derived from BM-MSCs and CESCs in the chondrogenic medium after 3 weeks. Pellets derived from BM-MSCs (A, B) and CESCs (C, D) stained with alcian blue (A, C) and toluidine blue (B, D). Immunohistochemistry for aggrecan (E, G) and type II collagen (F, H) in the pellets derived from BM-MSCs (E, F) and CESCs (G, H). Bar = 100 µm.

**Figure 15:**
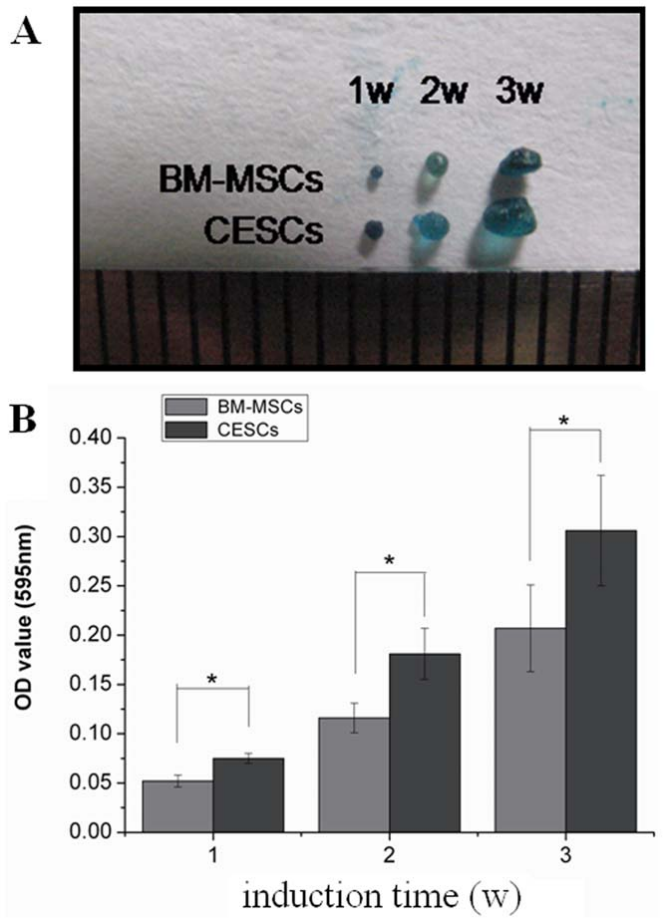
Alcian blue staining intensity of the micromass pellets from BM-MSCs and CESCs at corresponding time (week). A. Macro pictures of aggregates stained with alcian blue at week 1, 2 and 3. B. The absorbance of CESCs pellets was higher than that of BM-MSCs at each time point. * indicated p<0.05.

**Figure 16:**
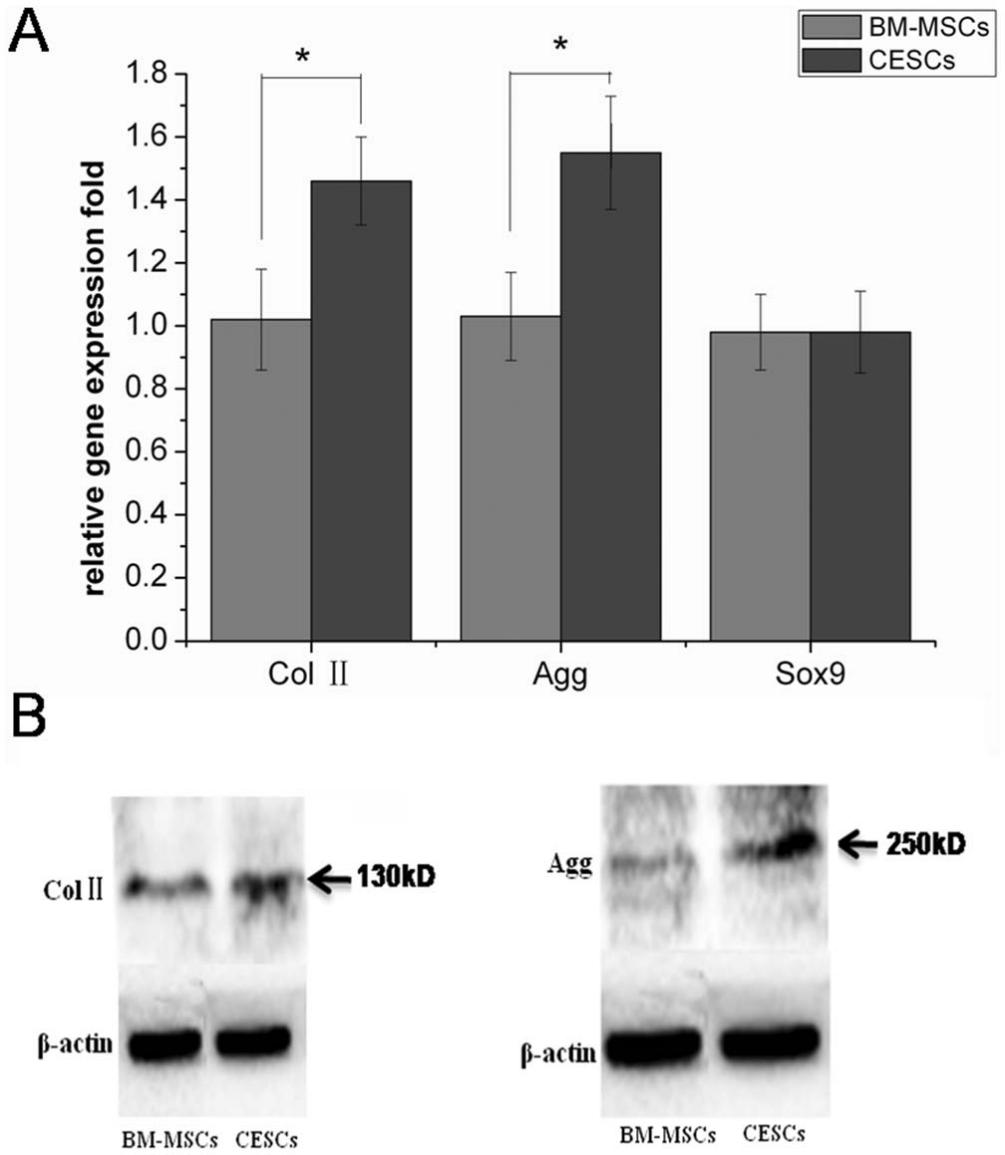
The assays of chondrogenesis derived from BM-MSCs and CESCs after 3 week culturing. A. The mRNA expression levels. There was a higher mRNA expression level of Col II and Agg in CESCs after 3 week induction (p<0.05). For SOX9, no significant difference was found. B. Western blots for Col II and aggrecan. Higher Col II and aggrecan protein levels could be found in CESCs. * indicated p<0.05.

For *in vitro* chondrogenesis, a micromass pellet culture was performed to evaluate the chondrogenic potential. BM-MSCs and CESCs were generated in the cellular nodules in the first 1–3 days, and they floated freely in the chondrogenic induction suspension culture medium. During chondrogenesis, the pellet size increased, and the pellets derived from CESCs were larger and heavier than the pellets from BM-MSCs at the corresponding induction time (Figure 13). Histologically, the cell pellets derived from the BM-MSCs and CESCs exhibited intense staining of sulfated proteoglycans after 3 weeks of induction (Figure 14). Immunohistochemical staining showed high accumulation of aggrecan and collagen II proteins in both pellets (Figure 14). The alcian blue staining intensity of the pellets derived from CESCs was higher than that of BM-MSCs at the corresponding time point (Figure 15). For the chondrogenic genes, the mRNA expression levels of *Col II* and *aggrecan* in CESCs were higher but no significant difference for *SOX9* was observed between the cell types (Figure 16A). Western blot analysis indicated that both Col II and aggrecan protein levels were higher in CESCs, which was in accordance with the qPCR results (Figure 16B). All of these results demonstrated that CESCs had a greater chondrogenesis potential than BM-MSCs.

## Discussion

In this study, we have successfully isolated a universal cell population with stem cell characteristics from degenerated human intervertebral disc CEP. This report is the first to isolate and characterize human CESCs *in vitro*. We have compared the morphology, proliferation potential, cell cycle, immunophenotype, stem cell gene expressions and differentiation ability of CESCs with those of BM-MSCs from the same subjects. The results reveal that CESCs are superior in terms of the osteogenic and chondrogenic capacity *in vitro*. This result demonstrates that CESCs may be a new cell source that may be more suitable for bone and cartilage repair.

The gross and histological morphology of the CEP showed that there were no vascular or fat tissues in the retrieved samples ensuring that other potential sources of stem cells were excluded. Agarose suspension culture is a chondrocyte selective culture system, in which chondrocytes are the only cell type to survive apart from tumor cells [27]. Thornemo cultured chondrocytes in agarose for six weeks and identified cell clusters with diameters greater than 50 µm. Furthermore, parts of these cell clusters had multi-directional differentiation capacity [22]. Using an agarose culture, we excluded the existence of cell types other than chondrocytes and found that some CEP-derived cells formed cell clusters with diameters larger than 50 µm. These cell clusters were chosen and expanded *in vitro*. Further results demonstrated that CESCs shared similar characteristics with BM-MSCs. First, CESCs were enriched by allowing the cells to adhere to a plastic tissue culture flask. The adherent cells exhibited morphology and proliferation capacity similar to those of BM-MSCs. Second, the CESCs were positive for many stem cell markers (e.g. CD105, CD73, CD90, CD44, CD166, Stro-1 and CD133), and negative for the pan-monocytic antigen CD14, the hematopoietic stem cell marker CD34, the pan-B-cell marker CD19, the pan-hematopoietic marker CD45, and the class 2 HLA antigen HLA-DR. Finally, the CESCs exhibited differentiation potential into osteoblasts, adipocytes and chondrocytes under the appropriate induction conditions. According to the criteria used to define MSCs stated by the International Society for Cellular Therapy (ISCT) [28], our results fulfill the majority of the ISCT requirements for MSCs definition: *in vitro* adherence to plastic surfaces, immunophenotypic profile and multilineage differentiation capacity into osteogenic, adipogenic and chondrogenic lineages. Additionally, CESCs and BM-MSCs share the same characteristics of cell cycle and stem cell genes. Reems et al. reported that the vast majority of primitive progenitor cells resided in the G0/G1 phase. Thus the percentage in the G0/G1 phase is more relevant to the stemness of cells [29]. In our study, the cell cycle results showed that the vast majority of both cell types were in the G0/G1phase and that less than 15% of the cells were in the S and G2+M phases. OCT-4, NANOG, and SOX-2, are transcription factors that play a central role in the regulation of pluripotency and self-renewal. These three transcription factors are expressed at high levels in pluripotent cells and are considered to be markers of primitive stem cells [30]. Expression of the above stem cell genes in our study provided more evidence for the existence of stem cells in the degenerated CEP.

Although CESCs share many characteristics with the BM-MSCs, other studies have indicated that variations existed among tissue sources [25], [31]. Our results are also in accordance with those findings. For the immunophenotypic profiles, the percentages of CD105- and CD166-positive cells in CESCs were lower compared to BM-MSCs. Three causes might explain this finding. First, variability in the epitope profile reflects the original tissue sources [32]. Second,digestion, isolation, different culturing conditions, or expansion *in vitro* may have resulted in changes to their original profiles [33]. Finally, these changes may be related to the function of the cell's origin. CD105 has been described as being related to hematopoiesis and cell migiration. CD166 is involved in homophilic and heterophilic adhesive interactions between early hematopoietic progenitors and associated stromal cells in primary blood-forming organs [34], [35]. Thus, these two adhesion molecules may be more important for BM-MSCs for homing capacity in the bone marrow.

Furthermore, our study indicated that the CESCs exhibited a higher osteogenic potential compared to the BM-MSCs. ALP activity is considered to be associated with osteoblastic differentiation, and it is thought to initially increase and then decrease when mineralization is well progressed [36], [37]. Our results were also in accordance with this model. For either BM-MSCs or CESCs, the mineralization extent gradually increased during the observation period. For both cell types, ALP activity increased in the first three weeks and then decreased in the fourth week. The qPCR results also demonstrated that CESCs had higher ALP expression levels, which is recognized as an early osteoblastic marker [38], and more osteocalcin, which is usually used as a marker of mature osteoblasts [39]. These results indicated that the osteogenic capacity of CESCs was superior in the early and late osteogenic stages.

No significant difference was detected regarding the adipogenic capacity of BM-MSCs and CESCs. However, the high DV variations observed in the BM-MSCs should benoted. This phenomenon may be a consequence of the heterogeneous cell population of the BM-MSCs [25]. In contrast, the CESCs used in the present study were derived from solid tissue and the cells were more homogeneous, which resulted in small DV variations.

Finally, we have quantitatively evaluated the chondrogenic potential of BM-MSCs and CESCs. Our results demonstrated that CESCs have a better chondrogenic ability than BM-MSCs according to the wet weight of micromass pellets, the dye absorbance of alcian blue and qPCR. Stable radioactivity per DNA in cells prelabeled with ^3^H-thymidine during *in vitro* chondrogenesis of MSCs indicated that there was little proliferation in the pellets. Thus, the pellet size was increased by the production of the extracellular matrix, not by the proliferation of the cells [40]. Therefore, the size or weight is considered to be a factor used to compare the chondrogenic potential of different stem cells [20]. For qPCR, the much stronger matrix production (collagen II and aggrecan) was not accompanied by an increase in *SOX-9* expression. *SOX-9* plays a critical role in chondrogenesis and stimulates the expression of extracellular matrix components, such as collagen II and aggrecan [41], [42]. Some authors believed that low *SOX-9* expression levels were already sufficient to support matrix production [43], [44]. In addition, the samples selected in the study were degenerated, and some authors believed that the expression of *SOX-9* decreased with increasing IVD degeneration grade [45].

Despite the strength of these findings, an inevitable problem must be addressed in our findings. The samples used in our study were degenerated; therefore, the biochemical composition within the degenerated CEP must have changed to some extent. The interactions between stem cells and their surrounding niche are essential to modulate cell morphology, phenotype and function, including *in vitro* differentiation [46], [47]. Therefore, the effect of biochemical changes in degenerated CEP on CESCs remains to be elucidated. Furthermore, the degenerated CEP might involve a substantial amount of inflammation factors and cytokines. The effects these mediators might have on the stem cells within the degenerated CEP are unknown. Whether the difference of immunophenotype and *in vitro* trilineage differentiation capacity between BM-MSCs and CESCs are related to the above causes needs to be identified.

In conclusion, this paper is the first report on the presence of stem cells in degenerated human intervertebral disc CEP, and we compared CESCs to BM-MSCs. Based on our results, the investigation into the characteristics of CESCs may improve our understanding of IVD pathophysiology and the degeneration process, and it would provide cell candidates for cell-based regenerative medicine and tissue engineering. In our future studies, we will investigate whether these cells can maintain their multipotency *in vivo* and in bioscaffolds, and whether they can be applied in tissue-engineering and regenerative medicine.
